# Improving venous thromboembolism risk assessments on an older age psychiatric ward – a complete audit cycle

**DOI:** 10.1192/bjo.2021.264

**Published:** 2021-06-18

**Authors:** Syazana JD, Edward Hart, Ranjit Mahanta, Alison Marshall

**Affiliations:** 1 Surrey and Borders Partnership NHS Foundation Trust; 2 Kingston Hospital NHS Foundation Trust

## Abstract

**Aims:**

Venous thromboembolism (VTE) is a common disease amongst hospital patients. Within acute hospitals, there are well established protocols for risk assessment and prevention of VTE via mechanical and pharmacological prophylaxes.

In psychiatry, assessment of VTE risk is more commonly overlooked despite many inherent risk factors which are unique to acute psychiatric admissions; including antipsychotic medications, physical restraint, catatonic states, and poor nutritional and hydration status[1]. The risk is compounded in older adult psychiatric patients, in which both patient and admission-related risk factors can act synergistically.

Anecdotally, it was reported that VTE assessments were not being completed and documented on the electronic patient record system. Our aim was to introduce a physical VTE risk assessment to attach to paper drug charts, which would act as a prompt for junior doctors, and serve to increase rates of completion.

**Method:**

A baseline retrospective audit of all patients admitted to the older adult inpatient ward over an 11-week period (05/08/2019~20/10/2019) was undertaken. The number of completed electronic VTE risk assessments at admission, and at 24 hours post-admission were calculated.

Subsequently, a new paper VTE risk assessment proforma was developed, combining the Department of Health VTE risk assessment tool[3] with several VTE risk factors associated with psychiatric patients (catatonia, antipsychotic medication, reduced oral intake, psychomotor retardation). Education was provided to the ward doctors, and regular assessments of VTE risk was incorporated into the weekly MDT meetings.

A re-audit was completed to assess the completion rates of the new paper VTE proforma. A snapshot style audit of all inpatients on the ward on Thursday 24th February 2020 was performed.

**Result:**

The baseline audit included 23 patients admitted during the 11-week period, consisting of 21 men and two women. The mean age was 74 years. Three patients (13% of total admissions) had their VTE and bleeding risk assessed on admission.

Following the implementation of a new VTE risk assessment proforma, the re-audit showed that all 19 inpatients (100% of total admissions) had a completed assessment. Although none of the patients required mechanical prophylaxis, one patient was receiving ongoing treatment for pulmonary embolism.

**Conclusion:**

VTE is a preventable disease, which historically has been under-recognised by psychiatric doctors. The introduction of a paper risk assessment proforma increased completion from 13% to 100%. It also prompted regular review of VTE risk during the weekly MDT meetings. This intervention may reduce the incidence of VTE-related pathology on the ward.
